# Associations between smoking, components of metabolic syndrome and lipoprotein particle size

**DOI:** 10.1186/1741-7015-11-195

**Published:** 2013-09-03

**Authors:** Sandra N Slagter, Jana V van Vliet-Ostaptchouk, Judith M Vonk, H Marike Boezen, Robin PF Dullaart, Anneke C Muller Kobold, Edith J Feskens, André P van Beek, Melanie M van der Klauw, Bruce HR Wolffenbuttel

**Affiliations:** 1 Department of Endocrinology, University of Groningen, University Medical Center Groningen, HPC AA31, PO Box 30001, Groningen 9700 RB, The Netherlands; 2 Department of Epidemiology, University of Groningen, University Medical Center Groningen, HPC AA31, PO Box 30001, Groningen 9700 RB, The Netherlands; 3 Department of Laboratory Medicine, University of Groningen, University Medical Center Groningen, HPC AA31, PO Box 30001, Groningen 9700 RB, The Netherlands; 4 Division of Human Nutrition, Wageningen University, PO Box 8129, Wageningen 6700 EV, The Netherlands

**Keywords:** Metabolic syndrome, Smoking, HDL, Cholesterol, Apolipoproteins, Triglycerides, Obesity, Cross-sectional, BMI classes

## Abstract

**Background:**

The clustering of metabolic and cardiovascular risk factors is known as metabolic syndrome (MetS). The risk of having MetS is strongly associated with increased adiposity and can be further modified by smoking behavior. Apolipoproteins (apo) associated with low-density lipoprotein-cholesterol (LDL-C) and high-density lipoprotein-cholesterol (HDL-C) may be altered in MetS. This study aimed to examine the association between smoking and the following parameters: MetS and its components, levels of apolipoproteins and estimated lipoprotein particle size, separately for men and women, and in different body mass index (BMI) classes.

**Methods:**

We included 24,389 men and 35,078 women aged between 18 and 80 years who participated in the LifeLines Cohort Study between December 2006 and January 2012; 5,685 men and 6,989 women were current smokers. Participants were categorized into three different body mass index (BMI) classes (BMI <25; BMI 25 to 30; BMI ≥30 kg/m^2^). MetS was defined according to the National Cholesterol Education Program’s Adult Treatment Panel III (NCEP:ATPIII) criteria. Blood pressure, anthropometric and lipid measurements were rigorously standardized, and the large sample size enabled a powerful estimate of quantitative changes. The association between smoking and the individual MetS components, and apoA1 and apoB, was tested with linear regression. Logistic regression was used to examine the effect of smoking and daily tobacco smoked on risk of having MetS. All models were age adjusted and stratified by sex and BMI class.

**Results:**

Prevalence of MetS increased with higher BMI levels. A total of 64% of obese men and 42% of obese women had MetS. Current smoking was associated with a higher risk of MetS in both sexes and all BMI classes (odds ratio 1.7 to 2.4 for men, 1.8 to 2.3 for women, all *P* values <0.001). Current smokers had lower levels of HDL cholesterol and apoA1, higher levels of triglycerides and apoB, and higher waist circumference than non-smokers (all *P* <0.001). Smoking had no consistent association with blood pressure or fasting blood glucose. In all BMI classes, we found a dose-dependent association of daily tobacco consumption with MetS prevalence as well as with lower levels of HDL cholesterol, higher triglyceride levels and lower ratios of HDL cholesterol/apoA1 and, only in those with BMI <30, LDL cholesterol/apoB (all *P* <0.001).

**Conclusions:**

Smoking is associated with an increased prevalence of MetS, independent of sex and BMI class. This increased risk is mainly related to lower HDL cholesterol, and higher triglycerides and waist circumference. In addition, smoking was associated with unfavorable changes in apoA1 and apoB, and in lipoprotein particle size.

Please see related commentary: http://www.biomedcentral.com/1741-7015/11/196.

## Background

Metabolic syndrome (MetS) is a combination of unfavorable health factors including abdominal obesity, dyslipidemia, hypertension and glucose intolerance [1,2] and is strongly associated with increased risk of cardiovascular disease (CVD) and type 2 diabetes [1,2]. One of the key drivers in the development of MetS is obesity [3]. In recent years, the global prevalence of obesity has increased at alarming rates, and MetS and its consequences have become a major public health burden [4,5]. This rise in MetS prevalence has also been observed in non-obese individuals [6-8] and there is strong evidence that the increase is mainly the result of unfavorable lifestyle changes, such as inactivity and poor nutrition [9].

Smoking has also been implicated as a risk factor for MetS. Earlier studies have suggested that overall tobacco use is associated with an increased risk of MetS [10,11], most likely due to its effects on waist circumference, blood lipids and blood pressure [10,12,13]. Such metabolic abnormalities may also be modulated by a direct negative effect of smoking on insulin resistance [12]. The degree to which smoking modulates the risk of developing obesity-related MetS still remains unclear, however. While the association between smoking, metabolic disturbances and the presence of MetS has been firmly established in obese individuals [7,8], with a similar trend observed in normal weight individuals [7], these findings could not be confirmed by others [8,14].

Alterations in the size and composition of low-density lipoprotein (LDL) particles and high-density lipoprotein (HDL) particles have been associated with metabolic syndrome [15], and are known to be related to CVD risk [16]. Individuals with altered HDL cholesterol (HDL-C) and triglyceride levels, two components of MetS, are more likely to also have unfavorable changes in the levels of apolipoproteins (apo) A1 and B, the apolipoproteins associated with HDL-C and LDL-C, as well as altered size and composition of these lipoprotein particles [17]. Although small-scale studies have suggested that smoking may influence the levels of apolipoproteins and the composition of lipoproteins [18-20], the extent to which this is associated with prevalent BMI and the risk of MetS is largely unknown. In addition, the latter studies have been published over two decades ago, and since then improved standardization has made apolipoprotein assays more reliable and reproducible [21,22].

The aim of the present study was to examine the association between smoking and the individual components of MetS in normal weight, overweight and obese subjects, in a very large population-based cohort study [23]. We also assessed the relationship between smoking and apolipoprotein levels, and between smoking and lipoprotein particle size, using the HDL-C/apoA1 and LDL-C/apoB ratios as a proxy.

## Methods

### Study design and subjects

The LifeLines Cohort Study is a multidisciplinary prospective population-based cohort study that examines the health and health-related behaviors of participants living in the northeast of The Netherlands [23]. It employs a wide range of procedures to assess the biomedical, sociodemographic, behavioral, physical and psychological factors that contribute to the health and disease of the general population, with a focus on multimorbidity. All participants filled in an extensive questionnaire about health-related items and lifestyle and underwent a clinical examination that included standard anthropometric and blood pressure measurements performed by trained technicians and collection of biological samples. All participants provided written informed consent before participating in the study. The study protocol was approved by the medical ethical review committee of the University Medical Center Groningen.

For this cross-sectional study we included subjects of Western European origin (according to self-reported information in the questionnaire), aged between 18 and 80 years who participated in the LifeLines Cohort Study between December 2006 and January 2012. Individuals who had missing data on BMI (n = 21), or on the variables needed to define MetS (n = 2,044), or whose questionnaires were incomplete with regard to smoking behavior (n = 2,202) were excluded from analysis. A total of 59,467 individuals were available for the current analysis.

### Clinical examination

The anthropometric measurements height, weight, waist and hip circumference, and blood pressure were conducted by trained technicians using a standardized protocol. Body weight was measured without shoes with 0.1 kg precision. Height, waist and hip circumference were measured to the nearest 0.5 cm. Waist circumference was measured in standing position with a tape measure all around the body, at the level midway between the lower rib margin and the iliac crest. Systolic and diastolic blood pressures were measured every minute for a period of 10 minutes using an automated Dinamap Monitor (GE Healthcare, Freiburg, Germany). The size of the cuff was chosen according to the arm circumference. The average of the last three readings was recorded for each blood pressure parameter.

### Biochemical measurements

At a second visit, blood was collected in the fasting state, between 8.00 and 10.00 a.m. The blood samples were transported under temperature-controlled conditions (at room temperature or at 4°C, depending on the sample requirements) to the LifeLines central laboratory facility. All measurements were performed the same day. Total and HDL cholesterol were measured using an enzymatic colorimetric method, triglycerides using a colorimetric UV method, and LDL-C using an enzymatic method, all on a Roche Modular P chemistry analyzer (Roche, Basel, Switzerland). Apolipoprotein A1 (apoA1) and apolipoprotein B (apoB) were measured by nephelometry (Siemens, Munich, Germany). Fasting blood glucose was measured using a hexokinase method.

### Assessment of metabolic syndrome and lipoprotein particle size

BMI was calculated as weight (kg) divided by height squared (m^2^). We classified the subjects into three BMI categories: normal weight (BMI <25.0), overweight (BMI 25.0 to 30) or obese (BMI ≥30). Individuals with a BMI <30 were considered to have MetS if they satisfied at least three of the five criteria named in the revised National Cholesterol Education Program’s Adult Treatment Panel III (NCEP:ATPIII, Table 1) [2]. Individuals with a BMI ≥30 were considered to have MetS if they satisfied at least two of the four MetS criteria (excluding waist circumference since a BMI ≥30 overrules the waist circumference criterion). The HDL-C/apoA1 ratio and LDL-C/apoB ratio were calculated to estimate differences in HDL-C and LDL-C particle size.

**Table 1:** **The revised National Cholesterol Education Program’s Adult Treatment Panel III criteria (NCEP:ATP III): for a person to be defined as having metabolic syndrome (MetS) they must satisfy at least three of the five criteria below**^**a**^

**Criteria**	**Details**
Raised blood pressure	Systolic blood pressure (SBP) ≥130 mmHg or diastolic blood pressure (DBP) ≥85 mmHg or use of blood pressure-lowering medication
Elevated glucose level	Fasting blood glucose ≥5.6 mmol/l or use of blood glucose-lowering medication or diagnosis of type 2 diabetes
Decreased high-density lipoprotein-cholesterol	<1.03 mmol/l in men or <1.30 mmol/l in women or lipid-lowering medical treatment
Elevated triglycerides	≥1.70 mmol/l or medication for elevated triglycerides
Abdominal obesity (increased waist circumference)	≥102 cm in men or ≥88 cm in women

^a^If body mass index (BMI) is ≥30 kg/m^2^, abdominal obesity can be assumed and waist circumference is not included as a criterion. A person with BMI ≥30 must satisfy at least two of the four other criteria to be defined as having MetS.

### Data description

Diagnosis of earlier myocardial infarction or hypertension was self-reported, as was the use of medication. Diagnosis of diabetes mellitus was based either on self-report, or on the finding of a fasting blood glucose >7 mmol/l. Information about smoking was collected from the self-administered questionnaires. Respondents were asked whether they smoked; whether they had smoked during the last month and whether they had ever smoked for an entire year; whether they had stopped smoking; which type of tobacco they currently smoked (cigarette, cigarillo, cigar, pipe tobacco or a mixture of different kinds); and the amount smoked (number of cigarettes smoked per day and/or grams tobacco per week, in the case of pipe smokers). The subjects were classified according to smoking status as non-smoker, former smoker or current smoker. Subjects were defined as a non-smoker if they had not smoked during the last month and had also never smoked for longer than a year. Former smokers were those who had not smoked during the last month but reported to have smoked for longer than a year and had stopped smoking. Current smokers were subjects who reported to have smoked during the last month or those who reported to have smoked for longer than a year and had not stopped smoking. Estimation of current smokers’ total tobacco use and their classification into light, moderate and heavy smokers were based on the following quantities: one cigarette = 1 g tobacco, one cigarillo = 3 g tobacco and one cigar = 5 g tobacco. Light smoking was defined as 10 g/day or less, moderate as 11 to 20 g/day and heavy as more than 20 g/day.

### Statistical methods

All analyses were conducted using IBM SPSS Statistics version 20 (IBM Corporation, Armonk, NY, USA). Data are presented as means ± SD, or geometric mean and interquartile range when they were not normally distributed. For comparisons between groups, analysis of variance was used where appropriate. Linear regression was used to examine the associations between smoking and the five components of MetS as well as between smoking and the apolipoprotein levels and the HDL-C/apoA1 and LDL-C/apoB ratios. Logistic regression was used to examine the effect of smoking and daily tobacco use on the risk of having MetS. This approach generated odds ratios that predicted the odds of having MetS for the different smoking statuses and different amounts of tobacco usage. Since distributions for triglyceride and fasting blood glucose were right skewed, before analysis we log-transformed (natural log) values to approximate normal distribution. All analyses were stratified for sex and BMI class, and were additionally adjusted for age. We applied a Bonferroni correction to account for the number of independent tests. A *P* value of ≤0.001 (0.05/48) was regarded as significant, given 48 independent tests (6 statistical models × 8 traits). Since the analyses were performed separately for men and women, and also for each BMI class, we used six models. The eight traits were as follows: (1) systolic and diastolic blood pressure or hypertension; (2) fasting glucose level; (3) HDL-C level; (4) triglyceride level; (5) waist circumference; (6) apoA1 and apoB; (7) HDL-C/apoA1 and LDL-C/apoB ratios; and (8) MetS.

## Results

The baseline characteristics of the participants are summarized in Table 2. Obesity prevalence was 14.4% in men and 16.1% in women. Subjects who were overweight or obese were slightly older than those with normal weight. Among normal weight men, 24.6% were current smokers, while 22.3% of the overweight and 23.1% of the obese were current smokers. Among normal weight women, 21.1% were current smokers, while 19.6% of the overweight and 16.8% of the obese were current smokers. For both sexes, systolic and diastolic blood pressure, serum triglycerides, blood glucose, LDL-C and apoB, as well as the percentage of subjects with type 2 diabetes, showed a consistent increase with increasing BMI. The same trend was observed for the percentage of subjects using medication to control elevated blood pressure, triglycerides or blood glucose. HDL-C and apoA1 levels, as well as the HDL-C/apoA1 ratio, showed a consistent decrease with increasing BMI. While in subjects with BMI <25 the overall prevalence of MetS was 3.6% in men and 2.4% in women, in the overweight this figure was 21.6% in men and 16.0% in women, rising to 64.3% of obese men and 41.5% of obese women.

**Table 2:** Characteristics of the current study population

**Characteristic**	**Men**				**Women**			
	**n = 24,389 (41.0%)**				**n = 35,078 (59.0%)**			
	**BMI <25**	**BMI 25 to 30**	**BMI ≥30**	***P*value**	**BMI <25**	**BMI 25 to 30**	**BMI ≥30**	***P*value**
n (%)	9,112 (37.4%)	11,763 (48.2%)	3,514 (14.4%)		17,750 (50.6%)	11,667 (33.3%)	5,661 (16.1%)	
Age, years	42 ± 12	47 ± 11	48 ± 11	<0.001	42 ± 12	47 ± 12	47 ± 12	<0.001
BMI, kg/m^2^	23.0 ± 1.5	27.1 ± 1.4	32.8 ± 2.9	<0.001	22.4 ± 1.7	27.1 ± 1.4	34.1 ± 3.9	<0.001
Smoking status								
Non-smoker, n (%)	4,467 (49.0%)	4,751 (40.4%)	1,311 (37.3%)		8,753 (49.3%)	5,080 (43.5%)	2,623 (46.3%)	
Former smoker, n (%)	2,401 (26.3%)	4,384 (37.3%)	1,390 (39.6%)		5,248 (29.6%)	4,298 (36.8%)	2,087 (36.9%)	
Current smoker, n (%)	2,244 (24.6%)	2,628 (22.3%)	813 (23.1%)		3,749 (21.1%)	2,289 (19.6%)	951 (16.8%)	
SBP, mmHg	127 ± 12	133 ± 13	137 ± 14	<0.001	119 ± 14	125 ± 15	130 ± 15	<0.001
DBP, mmHg	74 ± 8	78 ± 9	80 ± 9	<0.001	70 ± 8	73 ± 9	75 ± 9	<0.001
Total cholesterol, mmol/l	4.9 ± 1.0	5.2 ± 1.0	5.2 ± 1.0	<0.001	4.9 ± 1.0	5.1 ± 1.0	5.1 ± 1.0	<0.001
LDL-C, mmol/l	3.17 ± 0.86	3.47 ± 0.87	3.44 ± 0.91	<0.001	2.91 ± 0.84	3.25 ± 0.90	3.26 ± 0.88	<0.001
HDL-C, mmol/l	1.40 ± 0.32	1.25 ± 0.29	1.12 ± 0.26	<0.001	1.69 ± 0.39	1.54 ± 0.36	1.38 ± 0.33	<0.001
Triglycerides, mmol/l^a^	0.98 (0.71 to 1.31)	1.31 (0.91 to 1.80)	1.62 (1.15 to 2.23)	<0.001	0.81 (0.61 to 1.04)	0.99 (0.72 to 1.33)	1.20 (0.86 to 1.61)	<0.001
Apolipoprotein A1, g/l^b^	1.47 ± 0.23	1.41 ± 0.21	1.36 ± 0.21	<0.001	1.66 ± 0.28	1.60 ± 0.26	1.52 ± 0.26	<0.001
HDL-C/apoA1 ratio^b^	0.93 ± 0.13	0.86 ± 0.12	0.81 ± 0.11	<0.001	1.00 ± 0.14	0.95 ± 0.50	0.90 ± 0.49	<0.001
Apolipoprotein B, g/l^b^	0.91 ± 0.23	1.01 ± 0.24	1.05 ± 0.24	<0.001	0.84 ± 0.22	0.94 ± 0.24	0.97 ± 0.24	<0.001
LDL-C/apoB ratio^b^	3.51 ± 0.35	3.44 ± 0.40	3.32 ± 0.42	<0.001	3.45 ± 0.36	3.46 ± 0.36	3.37 ± 0.38	<0.001
Blood glucose, mmol/l^a^	4.94 (4.60 to 5.20)	5.18 (4.80 to 5.40)	5.52 (5.00 to 5.80)	<0.001	4.70 (4.40 to 4.90)	4.94 (4.60 to 5.20)	5.26 (4.80 to 5.50)	<0.001
Waist circumference, cm	87 ± 6	98 ± 6	112 ± 9	<0.001	79 ± 7	90 ± 7	105 ± 10	<0.001
BP-lowering medication, n (%)	462 (5.1%)	1,516 (12.9%)	843 (24.0%)	<0.001	1,050 (5.9%)	1,563 (13.4%)	1,351 (23.9%)	<0.001
Statin use, n (%)	267 (2.9%)	1,008 (8.6%)	473 (13.5%)	<0.001	364 (2.1%)	608 (5.2%)	457 (8.1%)	<0.001
TG-lowering medication, n (%)	6 (0.1%)	32 (0.3%)	13 (0.4%)	<0.001	3 (0.0%)	11 (0.1%)	8 (0.1%)	0.0012
Type 2 diabetes, n (%)	53 (0.6%)	203 (1.7%)	204 (5.8%)	<0.001	55 (0.3%)	150 (1.3%)	267 (4.7%)	<0.001
Oral antihyperglycemic medication, n (%)	38 (0.4%)	175 (1.5%)	175 (5.0%)	<0.001	44 (0.2%)	118 (1.0%)	211 (3.7%)	<0.001
Percentage fulfilling ≥3 out of 5 metabolic syndrome criteria^c^	330 (3.6%)	2,544 (21.6%)	2,259 (64.3%)	<0.001	425 (2.4%)	1,871 (16.0%)	2,351 (41.5%)	<0.001

Data are presented as mean ± SD, or median (interquartile range).

^a^Data given as geometric mean (interquartile range).

^b^ApoA1 and apoB results (and their ratios) were available in 34,613 and 34,601 of the 59,467 subjects, respectively.

^c^For subjects with BMI ≥30 kg/m^2^, two out of four criteria.

*Apo* apolipoprotein, *BMI* body mass index, *BP* blood pressure, *DBP* diastolic blood pressure, *HDL-C* high-density lipoprotein-cholesterol, *LDL-C* low-density lipoprotein-cholesterol, *SBP* systolic blood pressure, *TG* triglycerides.

For both sexes, former smokers were older and had higher levels of BMI, blood pressure, LDL-C, total cholesterol, waist circumference and glucose and were more frequently diagnosed with type 2 diabetes than non-smokers and current smokers (Table 3). Current smokers had the lowest levels of HDL-C and apoA1, the lowest HDL-C/apoA1 ratio, and the highest levels of triglycerides and, in women, apoB.

**Table 3:** Baseline characteristics of non-smokers, former smokers and current smokers

**Smoking status**	**Men**				**Women**			
	**Non-smoker**	**Former smoker**	**Current smoker**	***P*value**	**Non-smoker**	**Former smoker**	**Current smoker**	***P*value**
n (%)	10,529 (43.2%)	8,175 (33.5%)	5,685 (23.3%)		16,456 (46.9%)	11,633 (33.2%)	6,989 (19.9%)	
Age, years	42 ± 11	51 ± 12	43 ± 11	<0.001	43 ± 12	48 ± 11	42 ± 11	<0.001
BMI, kg/m^2^	26.1 ± 3.7	27.0 ± 3.5	26.2 ± 3.7	<0.001	25.6 ± 4.8	26.3 ± 4.7	25.4 ± 4.6	<0.001
BMI ≥30 kg/m^2^, n (%)	1,311 (12.5%)	1,390 (17.0%)	812 (14.3%)		2,623 (15.9%)	2,087 (17.9%)	951 (13.6%)	
SBP, mmHg	130 ± 13	133 ± 14	131 ± 13	<0.001	122 ± 15	124 ± 16	121 ± 14	<0.001
DBP, mmHg	76 ± 9	78 ± 9	76 ± 9	<0.001	72 ± 9	73 ± 9	72 ± 9	<0.001
Total cholesterol, mmol/l	5.0 ± 1.0	5.2 ± 1.0	5.1 ± 1.0	<0.001	4.9 ± 1.0	5.1 ± 1.0	5.0 ± 1.0	<0.001
LDL-C, mmol/l	3.28 ± 0.86	3.43 ± 0.89	3.39 ± 0.92	<0.001	2.99 ±0.85	3.18 ± 0.89	3.14 ± 0.92	<0.001
HDL-C, mmol/l	1.31 ± 0.31	1.31 ± 0.32	1.21 ± 0.30	<0.001	1.59 ± 0.38	1.65 ± 0.40	1.49 ± 0.38	<0.001
Triglycerides, mmol/l^a^	1.12 (0.78 to 1.54)	1.25 (0.87 to 1.73)	1.35 (0.92 to 1.91)	<0.001	0.87 (0.64 to 1.16)	0.93 (0.68 to 1.25)	1.01 (0.73 to 1.36)	<0.001
Apolipoprotein A1, g/l	1.43 ± 0.22	1.46 ± 0.22	1.40 ± 0.22	<0.001	1.61 ± 0.27	1.66 ± 0.27	1.57 ± 0.28	<0.001
HDL-C/apoA1 ratio	0.89 ± 0.13	0.88 ± 0.13	0.85 ± 0.13	<0.001	0.97 ± 0.14	0.98 ± 0.60	0.92 ± 0.13	<0.001
Apolipoprotein B, g/l	0.94 ± 0.23	1.00 ± 0.24	1.00 ± 0.25	<0.001	0.87 ± 0.23	0.91 ± 0.23	0.93 ± 0.24	<0.001
LDL-C/apoB ratio	3.49 ± 0.37	3.45 ± 0.41	3.37 ± 0.40	<0.001	3.44 ± 0.37	3.47 ± 0.36	3.38 ± 0.36	<0.001
Blood glucose, mmol/l^a^	5.05 (4.70 to 5.30)	5.26 (4.90 to 5.50)	5.11 (4.70 to 5.40)	<0.001	4.83 (4.50 to 5.10)	4.94 (4.60 to 5.20)	4.84 (4.50 to 5.10)	<0.001
Waist circumference, cm	94 ± 11	98 ± 10	95 ± 11	<0.001	86 ± 12	89 ± 12	87 ± 12	<0.001
BP-lowering medication, n (%)	821 (7.8%)	1,497 (18.3%)	503 (8.8%)	<0.001	1,683 (10.2%)	1,660 (14.3%)	621 (8.9%)	<0.001
Statin use, n (%)	411 (3.9%	918 (11.2%	419 (7.4%)	<0.001	560 (3.4%)	594 (5.1%)	275 (3.9%)	<0.001
TG-lowering medication, n (%)	13 (0.1%)	24 (0.3%)	14 (0.2%)	NS	6 (0.1%)	10 (0.1%)	6 (0.1%)	NS
Type 2 diabetes, n (%)	113 (1.1%)	264 (3.2%)	83 (1.5%)	<0.001	188 (1.1%)	216 (1.9%)	68 (1.0%)	NS
Oral antihyperglycemic medication, n (%)	102 (1.0%)	217 (2.7%)	69 (1.2%)	<0.001	154 (0.9%)	164 (1.4%)	55 (0.8%)	<0.001

Data are presented as mean ± SD, or median (interquartile range).

^a^Data given as geometric mean (interquartile range).

*Apo* apolipoprotein, *BMI* body mass index, *BP* blood pressure, *DBP* diastolic blood pressure, *HDL-C* high-density lipoprotein-cholesterol, *LDL-C* low-density lipoprotein-cholesterol, *NS* not significant, *SBP* systolic blood pressure, *TG* triglycerides.

**Figure 1:**
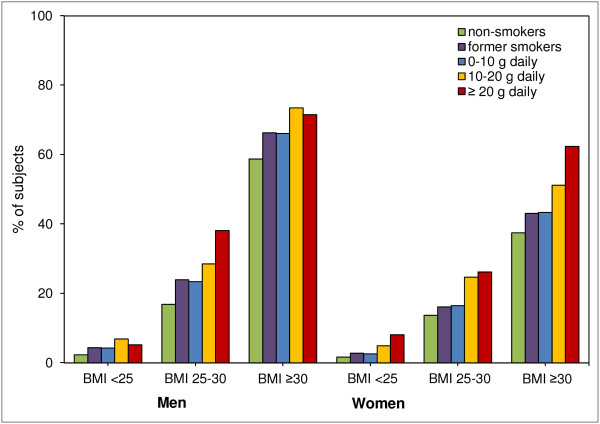
**Prevalence of metabolic syndrome in non-smokers, former smokers and current smokers.** Note that in all body mass index (BMI) classes prevalence of metabolic syndrome was higher in former smokers than in non-smokers, and that a dose–response relationship was found between prevalence of metabolic syndrome and amount of smoking, especially in women.

The percentage of subjects with MetS according to smoking status and daily tobacco consumption are shown in Figure 1. In both men and women, prevalence of MetS was greater in current smokers within each BMI group. In men, smoking was associated with higher MetS prevalence, although in the normal weight and obese men there was no difference between moderate and heavy smokers. In women there was a more pronounced dosage effect, that is, the percentage of individuals with MetS increased with an increase in the amount of tobacco smoked. Former smokers had a higher prevalence of MetS than non-smokers, but it should be taken into account that they were also older.

**Figure 2:**
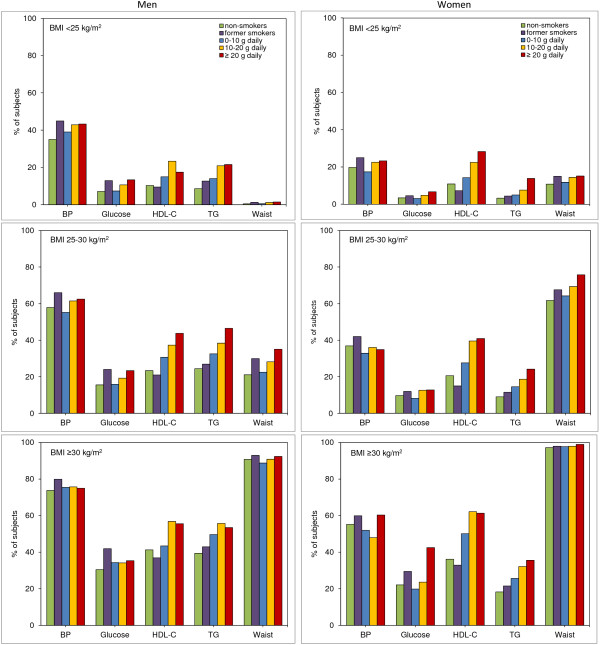
**Prevalence of the individual components of metabolic syndrome according to sex (left panel: men; right panel: women) and body mass index (BMI) class.** Top: BMI <25 kg/m^2^; middle; BMI 25 to 30 kg/m^2^; bottom: BMI ≥30 kg/m^2^. For all BMI classes, more men met the criteria for high blood pressure, elevated blood glucose and elevated triglyceride levels than did women, while women more frequently met the criteria for high waist circumference. Prevalence of high-density lipoprotein (HDL) abnormalities was not different between men and women. Higher tobacco consumption was particularly associated with abnormalities in HDL cholesterol and triglycerides, and to a lesser extent with abnormal waist circumference. BP, blood pressure; glucose, blood glucose; HDL-C, high-density lipoprotein-cholesterol; TG, triglycerides; waist, waist circumference.

For all BMI classes and smoking statuses, the percentage of subjects with high blood pressure, elevated blood glucose and elevated triglyceride levels was higher in men than in women, whereas women were more likely than men to have a higher waist circumference (Figure 2). In both sexes, increasing amounts of tobacco smoked were strongly associated with an increase in the number of individuals showing abnormal HDL-C and triglyceride levels. The amount of tobacco smoked was also associated with increased waist circumference, especially in overweight individuals. There were no consistent effects of the amount of tobacco smoked on blood pressure and blood glucose, nor did the amount of tobacco smoked influence blood pressure levels following correction for use of blood pressure-lowering medication.

Table 4 presents the associations between smoking and individual MetS components and between smoking and apolipoprotein levels and ratios, for the three different BMI classes, stratified by sex. There was a significant fall of HDL-C levels associated with greater amount of tobacco smoked in both sexes and all three BMI classes (*P* <0.001). In addition, the HDL-C/apoA1 ratio was significantly lower for higher amount of tobacco smoked in all BMI classes, and the LDL-C/apoB ratio for the lowest BMI classes (*P* <0.001). Former smokers had similar HDL-C levels to those of non-smokers. In all BMI classes, there was a consistent positive association between tobacco use and triglyceride levels (all *P* values <0.001). In all tobacco use groups, waist circumference was higher than that of non-smokers, independent of sex and BMI class, except for obese male light smokers. In obese female smokers we observed the largest rise in waist circumference: from 2.2 cm in moderate smokers to 6.4 cm in heavy smokers (both *P* <0.001). Moderate and heavy smoking was not associated with any strong changes in fasting blood glucose level.

**Table 4:** Effects of daily tobacco smoked on the components of MetS assessed by linear regression

**Component**	**Current smoking**					
	**Men**			**Women**		
	**Light**	**Moderate**	**Heavy**	**Light**	**Moderate**	**Heavy**
SBP, mmHg						
BMI <25	1.24 (0.46 to 2.03)	1.93 (1.10 to 2.76)	1.46 (−0.25 to 3.18)	−0.64 (−1.25 to −0.03)	0.22 (−0.51 to 0.95)	−0.01 (−1.73 to 1.71)
	NS	*P***<0.001**	NS	NS	NS	NS
BMI 25 to 30	−0.64 (−1.43 to 0.15)	1.59 (0.73 to 2.45)	0.21 (−1.30 to 1.72)	−1.46 (−2.30 to −0.61)	−0.06 (−1.05 to 0.92)	−1.13 (−3.37 to 1.11)
	NS	*P***<0.001**	NS	*P =***0.001**	NS	NS
BMI ≥30	0.58 (−1.08 to 2.25)	0.65 (−1.02 to 2.31)	2.16 (−0.24 to 4.55)	−0.93 (−2.39 to 0.53)	−0.74 (−2.24 to 0.77)	2.04 (−0.83 to 4.91)
	NS	NS	NS	NS	NS	NS
DBP, mmHg						
BMI <25	0.44 (−0.07 to 0.95)	0.94 (0.40 to 1.49)	1.68 (0.56 to 2.81)	−0.08 (−0.46 to 0.30)	0.49 (0.04 to 0.94)	1.13 (0.06 to 2.19)
	NS	*P =***0.001**	NS	NS	NS	NS
BMI 25 to 30	−0.38 (−0.91 to 0.15)	0.66 (0.08 to 1.23)	0.49 (−0.51 to 1.50)	−0.27 (−0.79 to 0.25)	0.56 (0.05 to 1.16)	0.66 (−0.72 to 2.04)
	NS	NS	NS	NS	NS	NS
BMI ≥30	−0.54 (−1.61 to 0.54)	0.35 (−0.71 to 1.42)	0.22 (−1.32 to 1.76)	−0.24 (−1.12 to 0.65)	−0.52 (−1.44 to 0.39)	0.86 (−0.88 to 2.59)
	NS	NS	NS	NS	NS	NS
HDL-C, mmol/l						
BMI <25	−0.07 (−0.09 to −0.05)	−0.13 (−0.15 to −0.11)	−0.14 (−0.18 to −0.09)	−0.06 (−0.08 to −0.05)	−0.17 (−0.20 to −0.15)	−0.21 (−0.26 to −0.16)
	*P***<0.001**	*P***<0.001**	*P***<0.001**	*P***<0.001**	*P***<0.001**	*P***<0.001**
BMI 25 to 30	−0.04 (−0.06 to −0.02)	−0.10 (−0.12 to −0.08)	−0.13 (−0.16 to −0.09)	−0.06 (−0.08 to −0.04)	−0.17 (−0.20 to −0.15)	−0.15 (−0.20 to −0.09)
	*P***<0.001**	*P***<0.001**	*P***<0.001**	*P***<0.001**	*P***<0.001**	*P***<0.001**
BMI ≥30	−0.04 (−0.07 to 0.01)	−0.10 (−0.13 to −0.07)	−0.10 (−0.14 to −0.06)	−0.08 (−0.12 to −0.05)	−0.17 (−0.20 to −0.13)	−0.20 (−0.26 to −0.14)
	NS	*P***<0.001**	*P***<0.001**	*P***<0.001**	*P***<0.001**	*P***<0.001**
Triglycerides, mmol/l^a^						
BMI <25	0.13 (0.10 to 0.15)	0.26 (0.23 to 0.29)	0.29 (0.22 to 0.37)	0.08 (0.06 to 0.09)	0.18 (0.16 to 0.19)	0.26 (0.21 to 0.31)
	*P***<0.001**	*P***<0.001**	*P***<0.001**	*P***<0.001**	*P***<0.001**	*P***<0.001**
BMI 25 to 30	0.16 (0.13 to 0.20)	0.30 (0.25 to 0.34)	0.40 (0.31 to 0.50)	0.11 (0.09 to 0.13)	0.21 (0.18 to 0.24)	0.33 (0.25 to 0.41)
	*P***<0.001**	*P***<0.001**	*P***<0.001**	*P***<0.001**	*P***<0.001**	*P***<0.001**
BMI ≥30	0.14 (0.07 to 0.22)	0.32 (0.22 to 0.41)	0.35 (0.20 to 0.53)	0.11 (0.07 to 0.16)	0.26 (0.23 to 0.31)	0.33 (0.23 to 0.46)
	NS	*P***<0.001**	*P***<0.001**	*P***<0.001**	*P***<0.001**	*P***<0.001**
Blood glucose, mmol/l^a^						
BMI <25	0.02 (0.01 to 0.04)	0.07 (0.04 to 0.10)	0.13 (0.05 to 0.21)	−0.01 (−0.02 to 0.01)	0.05 (0.03 to 0.08)	0.15 (0.08 to 0.24)
	NS	*P***<0.001**	NS	NS	NS	*P***<0.001**
BMI 25 to 30	0.01 (−0.02 to 0.03)	0.07 (0.03 to 0.11)	0.10 (0.03 to 0.16)	−0.03 (−0.05 to −0.01)	0.06 (0.03 to 0.10)	0.06 (−0.01 to 0.13)
	NS	*P =***0.001**	NS	NS	*P***<0.001**	NS
BMI ≥30	0.01 (−0.06 to 0.10)	0.04 (−0.05 to 0.13)	0.10 (−0.05 to 0.30)	−0.07 (−0.12 to −0.01)	0.05 (−0.01 to 0.13)	0.32 (0.17 to 0.50)
	NS	NS	NS	NS	NS	*P***<0.001**
Waist circumference, cm						
BMI <25	0.66 (0.27 to 1.05)	0.81 (0.39 to 1.23)	1.20 (0.34 to 2.06)	0.30 (−0.03 to 0.621)	0.83 (0.45 to 1.22)	1.18 (0.26 to 2.09)
	*P =***0.001**	*P***<0.001**	NS	NS	*P***<0.001**	NS
BMI 25 to 30	0.16 (−0.21 to 0.53)	1.53 (1.13 to 1.93)	1.99 (1.28 to 2.69)	0.74 (0.30 to 1.17)	1.75 (1.24 to 2.25)	1.61 (0.46 to 2.76)
	NS	*P***<0.001**	*P***<0.001**	*P =***0.001**	*P***<0.001**	NS
BMI ≥30	−0.97 (−2.05 to 0.11)	−0.06 (−1.14 to 1.01)	2.44 (0.89 to 3.99)	−0.04 (−1.10 to 1.01)	2.20 (1.10 to 3.29)	6.44 (4.37 to 8.51)
	NS	NS	NS	NS	*P***<0.001**	*P***<0.001**
Apo A1, g/l						
BMI <25	−0.02 (−0.04 to 0.00)	−0.05 (−0.06 to −0.03)	−0.04 (−0.08 to −0.00)	−0.00 (−0.02 to 0.02)	−0.06 (−0.08 to −0.04)	−0.07 (−0.12 to −0.02)
	NS	*P***<0.001**	NS	NS	*P***<0.001**	NS
BMI 25 to 30	−0.02 (−0.03 to 0.00)	−0.03 (−0.05 to −0.01)	−0.04 (−0.07 to −0.01)	−0.03 (−0.05 to −0.01)	−0.07 (−0.09 to −0.04)	−0.02 (−0.07 to 0.03)
	NS	*P***<0.001**	NS	NS	*P***<0.001**	NS
BMI ≥30	−0.02 (−0.05 to 0.02)	−0.04 (−0.08 to −0.01)	−0.03 (−0.07 to −0.02)	−0.07 (−0.10 to −0.03)	−0.09 (−0.13 to −0.06)	−0.09 (−0.16 to −0.03)
	NS	NS	NS	*P***<0.001**	*P***<0.001**	NS
HDL-C/apoA1 ratio						
BMI <25	−0.03 (−0.04 to −0.02)	−0.06 (−0.07 to −0.05)	−0.06 (−0.08 to −0.03)	−0.03 (−0.04 to −0.03)	−0.06 (−0.07 to −0.06)	−0.09 (−0.11 to −0.06)
	*P***<0.001**	*P***<0.001**	*P***<0.001**	*P***<0.001**	*P***<0.001**	*P***<0.001**
BMI 25 to 30	−0.02 (−0.03 to −0.01)	−0.05 (−0.06 to −0.04)	−0.06 (−0.08 to −0.05)	−0.04 (−0.05 to −0.03)	−0.06 (−0.07 to −0.05)	−0.06 (−0.09 to −0.04)
	*P***<0.001**	*P***<0.001**	*P***<0.001**	*P***<0.001**	*P***<0.001**	*P***<0.001**
BMI ≥30	−0.02 (−0.03 to 0.00)	−0.04 (−0.06 to −0.03)	−0.06 (−0.08 to −0.03)	−0.01 (−0.03 to 0.01)	−0.06 (−0.08 to −0.04)	−0.06 (−0.1 to −0.03)
	NS	*P***<0.001**	*P***<0.001**	NS	*P***<0.001**	*P =***<0.001**
Apo B, g/l						
BMI <25	0.03 (0.01 to 0.05)	0.09 (0.07 to 0.11)	0.09 (0.05 to 0.13)	0.03 (0.02 to 0.04)	0.09 (0.08 to 0.11)	0.14 (0.11 to 0.18)
	NS	*P***<0.001**	*P***<0.001**	*P***<0.001**	*P***<0.001**	*P***<0.001**
BMI 25 to 30	0.03 (0.02 to 0.05)	0.08 (0.06 to 0.10)	0.09 (0.06 to 0.12)	0.05 (0.03 to 0.06)	0.08 (0.06 to 0.10)	0.08 (0.04 to 0.13)
	*P***<0.001**	*P***<0.001**	*P***<0.001**	*P***<0.001**	*P***<0.001**	*P***<0.001**
BMI ≥30	0.02 (−0.02 to 0.07)	0.06 (0.02 to 0.09)	0.07 (0.02 to 0.12)	0.06 (0.03 to 0.09)	0.09 (0.06 to 0.12)	0.12 (0.06 to 0.17)
	NS	NS	NS	*P***<0.001**	*P***<0.001**	*P***<0.001**
LDL-C/apoB ratio						
BMI <25	−0.11 (−0.14 to −0.08)	−0.13 (−0.16 to −0.10)	−0.09 (−0.15 to −0.03)	−0.06 (−0.08 to −0.04)	−0.07 (−0.10 to −0.05)	−0.11 (−0.17 to −0.05)
	*P***<0.001**	*P***<0.001**	NS	*P***<0.001**	*P***<0.001**	*P =***0.001**
BMI 25 to 30	−0.08 (−0.12 to −0.06)	−0.12 (−0.15 to −0.09)	−0.19 (−0.24 to −0.13)	−0.05 (−0.07 to −0.02)	−0.06 (−0.09 to −0.02)	−0.13 (−0.20 to −0.06)
	*P***<0.001**	*P***<0.001**	*P***<0.001**	NS	*P =***0.001**	*P***<0.001**
BMI ≥30	−0.06 (−0.12 to 0.01)	−0.13 (−0.20 to −0.07)	−0.14 (−0.23 to −0.05)	−0.02 (−0.07 to 0.03)	−0.07 (−0.13 to −0.02)	−0.08 (−0.18 to 0.02)
	NS	*P =***<0.001**	NS	NS	NS	NS

Data are presented as mean effect size (95% confidence interval) per unit of component of metabolic syndrome or associated risk factor. Non-smokers within the same BMI class were taken as reference group. See Table 3 for number of subjects per group. Daily tobacco smoked: ≤10 g (light smoker), 11 to 20 g (moderate smoker), ≥20 g (heavy smoker). *P* values ≤0.001 are presented in bold.

^a^Data are presented as geometric mean effect size (95% confidence interval) per unit of component of metabolic syndrome.

*Apo* apolipoprotein, *BMI* body mass index, *BP* blood pressure, *DBP* diastolic blood pressure, *HDL-C* high-density lipoprotein-cholesterol, *LDL-C* low-density lipoprotein-cholesterol, *NS* not significant, *SBP* systolic blood pressure, *TG* triglycerides.

**Figure 3:**
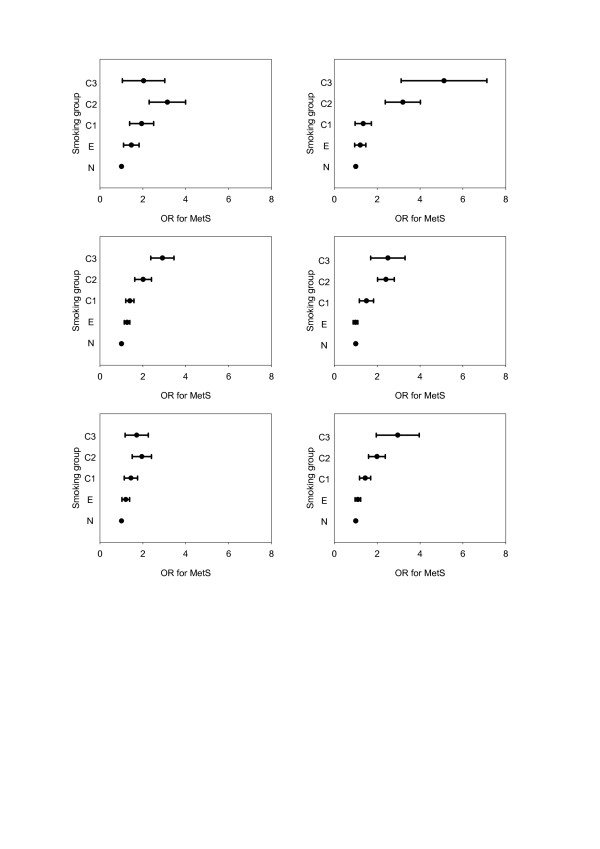
**Age-corrected odds ratios for having metabolic syndrome, in men (left panels) and women (right panels) according to body mass index (BMI) class.** N, non-smokers; E, former smokers; C1, smokers of 0 to 10 g tobacco daily; C2, smokers of 10 to 20 g daily; C3, smokers of ≥20 g daily. Top: BMI <25; middle: BMI 25 to 30; bottom: BMI ≥30 kg/m^2^.

The age-corrected odds ratios for having MetS, for men and women separately, in the three BMI classes, are depicted in Figure 3. In all BMI classes there was a significant rise in odds ratio with increasing amount of tobacco smoked. This trend was stronger in women than in men (*P* <0.001).

## Discussion

In the present study, performed in a large population-based cohort of almost 60,000 individuals, we investigated the relationship between smoking and the individual components of metabolic syndrome, and the association between smoking and levels of apolipoproteins and estimated lipoprotein particle size. Such a comprehensive and large-scale analysis has not been performed to date. We demonstrated that in both men and women smoking is associated with a greater prevalence of MetS, irrespective of their BMI. The largest differences between current smokers and non-smokers were observed in the levels of HDL-C and triglycerides, and, to a lesser extent, in waist circumference. While there were no consistent associations between smoking status and either blood pressure or fasting blood glucose levels, there was a dose-dependent relationship between the amount of tobacco smoked and decreased HDL-C levels and increased triglyceride levels. We also found a clear dose-dependent association between the amount of tobacco smoked and reduced ratios of HDL-C/apoA1 and LDL-C/apoB. To our knowledge, we are the first to explore these associations between smoking and levels of apolipoproteins and lipoprotein particle size in such a large cohort of individuals, with rigorously standardized physical and laboratory measurements, while taking into account both sex and BMI levels.

Our analysis revealed that in both men and women the prevalence of MetS was higher in current smokers in each BMI group, than in the non-smokers within that BMI group. Several earlier small-scale studies have reported smoking to be associated with higher prevalence of MetS [24-27]. The positive dose–response relationship between the amount of tobacco smoked and the prevalence of MetS that we observed is also consistent with previous studies [10,13,26,28]. However, when BMI was included in our analysis, the odds ratio for having MetS was higher among normal weight smoking subjects than those with higher BMI (Figure 3). This is probably related to the initial lower risk of subjects in this BMI group. Since previous studies have shown an excess of visceral fat to be a major contributor to metabolic abnormalities, overweight and obesity are known to be highly associated with MetS [29], with already a high prevalence of MetS observed in the obese non-smokers.

With our approach we have been able to calculate precisely the effects of smoking on the lipid parameters. Our data unequivocally show that despite the fact that obese men and women have a lower mean HDL-C than non-obese, the effects of heavy smoking are similar in all three BMI groups, with a consistent 0.10 to 0.14 mmol/l lower HDL-C for smoking men, and 0.15 to 0.21 mmol/l lower HDL-C in smoking women, in all three BMI groups (Table 4). The fact that we found current smoking to be mainly associated with lower levels of HDL-C, higher levels of triglycerides and larger waist circumference than the non-smoking status is consistent with earlier cross-sectional studies [28,30]. This observation of a dose-dependent relationship between the daily amount of tobacco smoked and lower HDL-C and higher triglycerides confirms the results of previous reports [13,31-33]. In our study, the magnitude of the effects of tobacco usage on HDL-C varied between 0.04 for light smoking in men, and 0.21 mmol/l for heavy smoking in normal weight women. The study by Chen *et al*., comprising 1,164 men, reported a similar dose–response relationship with the largest effect on HDL-C and triglycerides seen in those who smoked more than 40 cigarettes per day [13]. Ishizaka *et al*. also reported a dose–response association between the number of cigarettes per day and prevalence of MetS in a cohort of 5,033 individuals, although they did not examine the influence of the amount of tobacco smoked on the individual MetS components [33]. A recent review summarized the effects of smoking cessation on HDL-C levels: within a few weeks after stopping smoking, HDL-C levels start to increase, resulting in an overall increase of 0.2 mmol/l [34]. Taken together, these and our data support the causal relationship between smoking and low HDL-C levels.

There are indications that current smoking is associated with increased abdominal obesity [35]. In our study, although current smokers had a greater waist circumference than non-smokers, these differences were rather small. We also observed a consistent increase in the waist circumference with an increase in tobacco smoked in normal weight and overweight men, as well as in normal weight and obese women. Larger effects were especially seen among obese women, where the increase in waist circumference was 2.2 cm for moderate smokers and 6.4 cm for heavy smokers. One of the possible mechanisms that might explain these observations is a direct effect of smoking on cortisol production [12,36]. Indeed, it was demonstrated more than three decades ago that smokers have higher fasting plasma cortisol levels than non-smokers [37,38]. The increase in cortisol production leads to accumulation of abdominal fat [39], which, in turn, increases waist circumference.

Although some studies have indicated that smoking is related to reduced insulin sensitivity and the development of insulin resistance [12,40] and type 2 diabetes [41,42], in our population there was no consistent association between smoking and fasting blood glucose. This confirms the results obtained in other studies [13,30,43]. Ishizaka *et al*. found a higher prevalence of elevated blood glucose in smoking men, but not in women [33]. Such discrepancy in the results may be due to the different cut-off values for elevated fasting glucose used in the present study (5.6 mmol/l) and that of Ishizaka *et al*. (6.1 mmol/l) [33].

While it is well established that acute smoking may cause a rise in blood pressure [44,45], in the chronic situation smokers’ blood pressure is similar to or even lower than that of non-smokers [33,44,46], although Primatesta *et al*. found higher blood pressure in male smokers older than 45 years compared to never smokers [47]. We found no association between smoking and blood pressure in any of the three BMI classes, even after correction for the use of blood pressure-lowering medication. In addition, we found similar blood pressure in smokers aged 45 and higher versus non-smokers (data not shown). Nevertheless, some studies have suggested that smoking may be a risk factor for developing hypertension [48] or for an increase in blood pressure during exercise [49], although in the latter study smoking cessation did not lead to reduced blood pressure. Weight changes after smoking cessation have been suggested to be involved in this paradox [48].

One of the new findings of our study is the association between smoking and alterations in levels of apolipoproteins and in the size of lipoprotein particles. Until now, only a limited number of studies have investigated the relationship between smoking and the levels of apoA1 and apoB, usually involving a small number of participants such as, for example, young adults [18,50], middle-aged men [19,51], or postmenopausal women [20]. In addition, few studies have assessed the effects of smoking on lipoprotein particle size. In the Framingham study, smoking was associated with higher levels of small LDL particles [52]. However, apoA1 and apoB measurement and standardization have considerably improved in the last decade, both because of the appearance of a legal and regulatory framework (the In Vitro Diagnostics (IVD)-directive 98/79/EC and the institution of the Joint Committee on Traceability in Laboratory Medicine (JCTLM)), technical improvements of equipment, and the availability of international reference materials [21]. An additional milestone was the preparation, evaluation and introduction of value-assigned reference materials for monitoring trueness of apolipoprotein test results [22]. ApoA1 is the main protein component of HDL-C particles, and higher levels of apoA1 are associated with lower risk of CVD [53]. We observed that in current smokers plasma apoA1 levels were lower than in non-smokers. In addition, smoking was associated with lower HDL-C/apoA1 ratio, which is a strong indication of smaller HDL particle size. Such alterations of the HDL particle have been negatively associated with heart disease [54,55]. While apoA1 is protective, apoB, the main protein component of LDL particles, reflects the atherogenic potential of LDL, and higher levels of apoB are associated with an increased risk of CVD [53]. The fact that we found higher apoB levels and lower LDL-C/apoB ratios in current smokers than in non-smokers, indicates the presence of increased numbers of small, more dense LDL particles. Such particles have been found to increase the risk not only of atherosclerosis [56,57], but also of coronary artery disease [58] and fatal myocardial infarction [59]. Furthermore, in a 3-year follow-up study among Korean men without MetS, a low LDL-C/apoB ratio was independently associated with development of MetS [60]. Taken together with our findings, the Korean study supports the conclusion that the presence of increased amounts of small, dense LDL particles can be considered both a risk factor for future cardiovascular disease and an early feature of metabolic syndrome.

Our study has several major strengths. Considering the number of participants recruited from the general population (N >59,000), this is the largest study reporting these results. Our large dataset also enabled to carefully calculate effect sizes, and to perform sufficiently powered subgroup analyses, in subjects of both sexes and in those with normal body weight, overweight, and obesity, which to our knowledge has never been performed before. All participants to the LifeLines Cohort Study have been well characterized, with rigorously standardized blood pressure and anthropometric measurements. In addition, all laboratory measurements of lipids and apolipoproteins have been carried out over a period of 5 years in fresh serum samples, in the same certified laboratory, with the same equipment, and the same rigorous quality assessment and control. This unprecedented sample size also provided us with sufficient statistical power to investigate contradictory associations reported previously.

There are also some limitations to our study. Firstly, since smoking status was based on self-administered questionnaires, we cannot exclude the possibility that misreporting led to some individuals being misclassified with regard to their current smoking status. Considering the large number of participants, we believe that misclassification has only very limited influence on the results obtained, and earlier studies also reported low misclassification rate of smoking status [61]. We should point out that we were unable to identify individuals who had never smoked, nor could we fully take into account the duration of smoking. Secondly, apart from age we could not adjust for other possibly relevant risk factors that influence levels of HDL cholesterol and triglycerides, such as nutrition and alcohol consumption. As data collection for the LifeLines Cohort Study is still ongoing, we hope to be able to investigate the effects of such factors on MetS in the future.

## Conclusions

In this very large study in individuals of western European descent, smoking was associated with an increased risk of MetS. This increased risk was observed in all BMI classes. The elevated risk of having MetS was mainly related to lower HDL cholesterol, higher triglycerides and larger waist circumference. We also found that smoking was associated with unfavorable changes in the levels of apoA1 and apoB and in estimated HDL and LDL particle size, thereby providing a new pathophysiological mechanism linking smoking to increased risk of cardiovascular disease.

## Abbreviations

Apo: Apolipoprotein; BMI: Body mass index; CVD: Cardiovascular disease; HDL: High-density lipoprotein; LDL: Low-density lipoprotein; MetS: Metabolic syndrome; TG: Triglycerides.

## Competing interests

The authors declare that they have no competing interests.

## Authors’ contributions

SNS, JVvVO and BHRW carried out the statistical analyses and drafted the manuscript. ACMK coordinated all laboratory measurements and immunoassays. MMvdK, JMV, EJF and BHRW participated in the design of the cohort study and data collection, while JMV, HMB, SNS and MMvdK carried out the data verification and validation. RPFD and APvB participated in the data interpretation. All authors participated in drafting the manuscript, and read and approved the final version.

## Pre-publication history

The pre-publication history for this paper can be accessed here:

http://www.biomedcentral.com/1741-7015/11/195/prepub
